# The effects of *DICER1* and *DROSHA* polymorphisms on susceptibility to recurrent spontaneous abortion

**DOI:** 10.1002/jcla.23079

**Published:** 2019-10-28

**Authors:** Marzieh Ghasemi, Mahnaz Rezaei, Atefeh Yazdi, Narjes Keikha, Rostam Maruei‐Milan, Mina Asadi‐Tarani, Saeedeh Salimi

**Affiliations:** ^1^ Pregnancy Health Research Center Zahedan University of Medical Sciences Zahedan Iran; ^2^ Moloud Infertility Center Ali ibn Abitaleb Hospital Zahedan University of Medical Sciences Zahedan Iran; ^3^ Department of Clinical Biochemistry School of Medicine Zahedan University of Medical Sciences Zahedan Iran; ^4^ Department of Obstetrics and Gynecology School of Medicine Zahedan University of Medical Sciences Zahedan Iran; ^5^ Cellular and Molecular Research Center Zahedan University of Medical Sciences Zahedan Iran

**Keywords:** *DICER1*, *DROSHA*, polymorphism, recurrent spontaneous abortion

## Abstract

**Background:**

Recurrent spontaneous abortion (RSA) is a serious problem in pregnancy. The exact etiology of RSA is unknown in more than 50% of all the patients. However, genetic variations are known as susceptibility factors for idiopathic RSA. Considering the role of miRNA biosynthesis machinery in the miRNA production and effect of miRNAs on various diseases, this study aimed to evaluate the effects of *DICER1* rs3742330 and *DROSHA* rs6877842 polymorphisms on RSA risk.

**Methods:**

In this case‐control study, 150 RSA patients and 195 age‐matched healthy female controls were recruited. Both polymorphisms were genotyped using PCR‐RFLP method.

**Results:**

The frequency of *DICER1* rs3742330AG genotype was higher in the control group (*P* = .022). There was a statistically significant association between rs3742330 polymorphism and a reduced RSA risk in dominant and allelic models (*P* = .013 and *P* = .007, respectively). No statistically significant association was found between *DROSHA* rs6877842 variant and RSA risk. The combination of AG and GC genotypes and G‐G alleles of *DICER1* rs3742330 and *DROSHA* rs6877842 polymorphisms led to a decreased RSA risk. However, the synergic effect of rs3742330A and rs6877842G alleles (A‐G) and AA‐GG genotypes was associated with an increased RSA risk.

**Conclusion:**

the *DICER1* rs3742330AG genotype and combination of AG and GC genotypes and G‐G alleles of *DICER1* rs3742330 and *DROSHA* rs6877842 polymorphisms were associated with a reduced RSA risk.

## 1 INTRODUCTION

Recurrent spontaneous abortion (RSA) is defined as the occurrence of two or more spontaneous abortions before 20th week of gestation. It is one of the most common complications of gestation and infertility which occurs in approximately 1 to 2 percent of pregnant women.^1^ The etiology of RSA is not well understood although chromosomal abnormalities including translocations, inversions, deletions, and duplications are known in 50% of first trimester spontaneous abortions.^2^ Moreover, uterine anatomic anomalies, metabolic and endocrine problems, abnormal inflammation responses, thrombophilia, and infections are identified as other risk factors for the pathogenesis of RSA.^3^, ^4^ However, the exact cause of RSA remains unknown in more than 50% of RSA cases classified as idiopathic unexplained RSA (URSA).^5^

Genetic variations are described as other risk factors for susceptibility to idiopathic RSA.^6^ Higher frequency of RSA in siblings of patients confirmed the genetic etiology for idiopathic RSA. In addition, similar gestational age of miscarriage in a mother is another reason for this theory.^7^

Recently, the role of noncoding RNAs like microRNAs (miRNA) in the different cellular processes, such as proliferation, differentiation, cell death, immunity, and metabolism, has been established. miRNAs are ~22 nucleotides noncoding RNAs which can affect gene expression at the post‐transcriptional level by translational suppression or mRNA degradation. These short RNAs are generated from primary transcripts by nuclease activity of various enzymes in several steps, called miRNA biosynthesis machinery.^8^, ^9^

In the first step, Drosha enzyme catalyzes the conversion of first transcript to precursor miRNA (pre‐miRNA) hairpins. In the second step, the transfer of pre‐miRNAs to the cytoplasm is done by Ran‐GTPase (Ran) and exportin 5 (Xpo5). Then, they are converted into small interfering RNA and microRNA by a Dicer1 enzyme. Therefore, insufficient function or level of these enzymes can lead to altered concentration of miRNAs and subsequently their target genes.^8^, ^9^ The altered levels of several miRNAs have been reported in RSA patients.^10^, ^11^

There is also evidence that enzymes of this process are involved in cell proliferation, differentiation, and apoptosis.^12^ Indeed, several studies have shown that that Dicer and other components in miRNA biogenesis play key roles in sex‐related activities for both females and males, including follicular development, ovulation, luteinization, sex hormone synthesis, and the regulation of the functions of the fallopian tube and endometrial receptivity in female reproduction.^13^

Considering the role of genetic variants in pathogenesis of multifactorial diseases, numerous studies have evaluated the effects of miRNA and miRNA biosynthesis machinery polymorphisms on their pathogenesis.^14^, ^15^, ^16^ *DROSHA* and *DICER1* genes are located on chromosomes 5 and 14, respectively.^17^, ^18^ There are several single nucleotide polymorphisms (SNPs) in *DROSHA* and *DICER1* genes, and their effects on various diseases have been investigated.^16^, ^19^

Two other studies demonstrated that the combination of *DROSHA* and *DICER1* polymorphisms could increase RSA risk.^20^, ^21^ To date, very few studies have been conducted on the effects of *DROSHA* and *DICER1* polymorphisms on RSA. Since no study in this regard has been done in Iran, the present study was designed to examine the effects of *DROSHA* and *DICER1* polymorphisms on RSA susceptibility in an Iranian population.

## 2 MATERIALS AND METHODS

One hundred and fifty RSA women attended to Ali ibn Abi Taleb Hospital were recruited from October 2018 to February 2019. The RSA women with a history of at least two consecutive miscarriages before 20 weeks of gestation who had no successful pregnancy were included in study. Moreover, they had no history of known factors affecting RSA, such as anatomical abnormalities (confirmed by hysterosalpingography), abnormal inflammation responses and autoimmune diseases, metabolic and endocrine problems (TSH, FSH, LH, and prolactin), thrombophilia, and infections. All RSA women and their partners had a normal karyotype.

The control group was composed of 195 age‐matched healthy women who had no history of spontaneous abortion and infertility at least one live birth. Moreover, controls had no history of autoimmune diseases, metabolic and endocrine complications, and any systemic disease.

All study participants gave written informed consent. The research was approved by the ethic committee (IR. ZAUMS. REC.1396.261).

### 2.1 Genotyping

Five hundred microliters of EDTA‐treated blood samples was collected for DNA extraction by salting out method. The restriction fragment length polymorphism (RFLP) was used for the genotyping of both polymorphisms.^22^, ^23^ The protocol for PCR digestion was performed as previously described.^14^, ^24^

### 2.2 Statistical analysis

Hardy‐Weinberg equilibrium was analyzed by chi‐square test in both groups. The odds ratio (OR) and 95% confidence interval (CI) were calculated using logistic regression analysis in order to assess the association between polymorphisms and RSA *P* < .05 was considered as statistically significant. Student's *t* test and Fisher's exact test were used for the comparison of two groups for quantitative and qualitative parameters. Statistical analysis was performed with SPSS version 23.

## 3 RESULTS

Table 1 summarizes the demographic and clinical data of patients with RSA and control females.

**Table 1:** The demographic and clinical data of RSA and control women

	Control n = 195	RSA n = 150	*P*‐value
Age (y)	30.9 ± 7.4	32.2 ± 6.6	.1
BMI (kg/m^2^)	25.2 ± 4.1	25.9 ± 4.8	.2
Parity	2.3 ± 1.3	3.2 ± 1.4	<.0001
Deliveries	2.3 ± 1.3	0	<.0001
Abortions	0	3.2 ± 1.4	<.0001
Gestational age at abortion (wk)	39.2 ± 1.9	7.7 ± 5.5	<.0001

**Figure 1:**
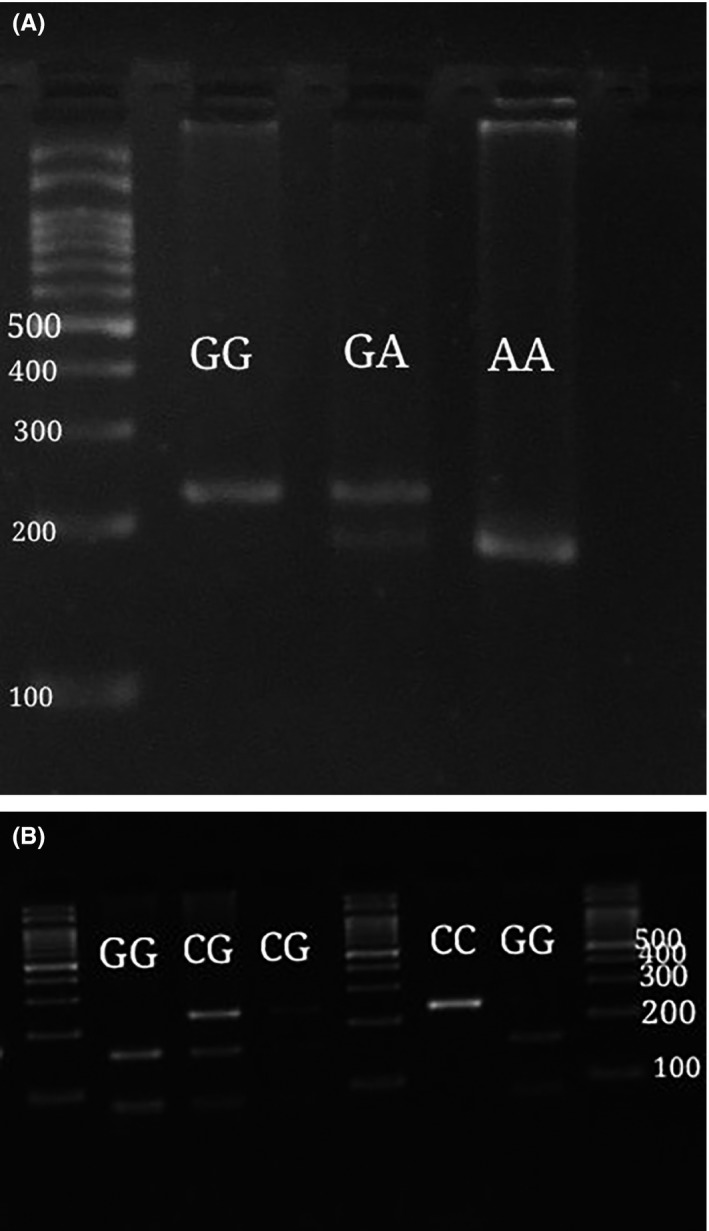
The agarose gel electrophoresis after digestion by A, *BanI* for *DICER1* rs3742330 polymorphism and B, *Sau96I* for *DROSHA* rs6877842 polymorphism

In addition, Figure 1 shows the results of agarose gel electrophoresis for *DICER1* rs3742330 and *DROSHA* rs6877842 polymorphisms using PCR‐RFLP method. The frequency of *DICER1* rs3742330 AG genotype in control group was higher than that in RSA women (26.7% vs 16.7%), associated with a 0.5‐fold increased risk of RSA (*P* = .022). Moreover, this variant might decrease RSA risk in dominant and allelic models. The frequencies of *DROSHA* rs6877842 GC and CC genotypes were higher in control group, but the differences were not significant (Table 2).

**Table 2:** Allelic and genotypic frequency of *DICER1* rs3742330 and *DROSHA* rs6877842 polymorphisms in recurrent spontaneous abortion women and control group

	RSA (n = 150)	Control (n = 195)	*P*‐value	OR (95% CI)
*DICER1 rs3742330*				
AA, n (%)	123 (82)	137 (70.3)		1
AG, n (%)	25 (16.7)	52 (26.7)	.022	0.5 (0.3‐0.9)
GG, n (%)	2 (1.3)	6 (3.1)	.230	0.4 (0.1‐1.2)
Dominant (AG + GG vs AA) Recessive (GG vs AG + AA)			.013	0.5 (0.3‐0.9)
			.300	0.4 (0.1‐2.1)
Allele				
A, n (%)	271 (90.3)	326 (83.6)		1
G, n (%)	29 (9.7)	64 (16.4)	.007	0.6 (0.3‐0.9)
*DROSHA rs6877842*				
GG, n (%)	126 (84)	149 (76.4)		1
GC, n (%)	21 (14)	41 (21)	.089	0.6 (0.3 ‐ 1.1)
CC, n (%)	3 (2)	5 (2.6)	.643	0.7 (0.2‐3)
Dominant (GC + CC vs GG) Recessive (CC vs GC + GG)			.084	0.6 (0.4‐1.1)
			.731	0.8 (0.2‐3.3)
Allele				
G, n (%)	273 (91)	339 (86.9)		1
C, n (%)	27 (9)	51 (13.1)	.06	0.7 (0.4‐1.1)

When we analyzed the effects of the combination of *DICER1* rs3742330 and *DROSHA* rs6877842 polymorphisms on RSA susceptibility, we found higher frequencies of AA/GG combined genotypes in RSA cases and all other combined genotypes in controls (*P* = .008, Table 3). Moreover, the AG/GC combined genotype was associated with a decreased risk of RSA (*P* = .03).

**Table 3:** Combination effects of *DICER1* rs3742330 *and DROSHA* rs6877842 polymorphisms on recurrent spontaneous abortion

*DICER1 rs3742330*	*DROSHA rs6877842*	Control n (%)	Case n (%)	*P*‐value	OR (95% CI)
AA	GG	103 (52.8)	101 (67.3)	‐	1
AA	GC	29 (14.9)	19 (12.7)	.22	0.7 (0.4‐1.3)
AA	CC	5 (2.6)	3 (2)	.51	0.6 (0.1‐2.6)
AG	GG	41 (21)	23 (15.3)	.06	0.6 (0.3‐1)
AG	GC	11 (5.6)	2 (1.3)	.03	0.2 (0.04‐0.9)
AG	CC	0	0	0	‐
GG	GG	5 (2.6)	2 (1.3)	.3	0.4 (0.1‐2.2)
GG	GC	1 (0.5)	0	‐	‐
GG	CC	0	0	‐	‐
AA	GG	103 (52.8)	101 (67.3)	.008	1.8 (1.2‐2.9)
Other genotypes		92 (47.2)	49 (32.7)	‐	‐

In addition, the analysis of synergic effects of *DICER1* rs3742330 and *DROSHA* rs6877842 alleles showed that the synergic presence of rs3742330A and rs6877842G alleles (A‐G) was more frequent in RSA patients, associated with a 1.7‐fold increased risk of RSA. But the G‐G condition could lead to a decreased RSA risk (Table 4).

**Table 4:** Combination effects of *DICER1* rs3742330 *and DROSHA* rs6877842 alleles on recurrent spontaneous abortion

	RSA n = 150	Control n = 195	*P*‐value	OR (95% CI)
A‐G	244 (81.3)	279 (71.5)	.003	1.7 (1.3‐2.5)
A‐C	27 (9)	47 (12.1)	.22	0.7 (0.4‐1.2)
G‐G	29 (9.7)	60 (15.4)	.03	0.6 (0.4‐0.9)
G‐C	0 (0)	4 (1)	**‐**	**‐**

## 4 DISCUSSION

miRNAs as a class of noncoding RNAs can regulate several processes in the placentas, such as placental immune activation and trophoblast invasion; therefore, they can affect gestational process.^25^ Aberrant expression and dysregulated pregnancy associated with miRNAs have been proven in several pregnancy complications, including preeclampsia and RSA.^25^, ^26^ It is well known that the disorder in miRNAs function or level can distress the expression levels of various target genes, which may affect numerous cellular functions. Evidences have shown the effects of several miRNAs on the regulation of cell proliferation and apoptosis, which can play key roles in critical processes of normal embryogenesis.^27^ Considering the important role of miRNAs in human placentas and abundant expression of them in this organ, growing evidences have identified the dysregulation of miRNAs in RSA pathogenesis.^28^

Several enzymes and proteins are involved in miRNAs synthesis and maturation, and RNA polymerase II, Drosha Ran‐GTPase, exportin 5, and Dicer are considered as one of the most important components. Considering the role of them in biogenesis of miRNAs, they are known as key molecules in various processes of normal pregnancy.^13^ Several other recent studies have evaluated the levels of miRNA machinery proteins in various pregnancy complications.^15^ Since a genetic etiology of RSA has been detected, numerous studies have examined the effects of genetic polymorphisms of miRNAs and miRNA machinery biogenesis genes on the pathogenesis of RSA.^10^, ^20^

In the current study, we evaluated the effects of *DICER1* rs3742330 and *DROSHA* rs6877842 polymorphisms on RSA and found a negative association between *DICER1* rs3742330 polymorphism and RSA risk in dominant and allelic models. However, we did not find any statistically significant relationship between *DROSHA* rs6877842 polymorphism and RSA in each model. The analysis of combination of the of *DICER1* rs3742330 and *DROSHA* rs6877842 polymorphisms on RSA showed that AG‐GC combined genotypes and G‐G haplotype were associated with a reduced RSA risk; however, AA‐GG combined genotypes and A‐G haplotype could lead to an increased RSA risk.

Dicer and Drosha are the two key enzymes in miRNA biogenesis. The aberrant expression of these enzymes is associated with various pregnancy complications. Evidence showed the effects of these enzymes on the reproductive functions for both males and females which their insufficiency could lead to RSA.^13^

A study conducted on human embryonic stem cells revealed the increased expression of Dicer during in vitro decidualization. The experimental analysis of mice with deleted Dicer in vascular smooth muscle cells showed that this defect could lead to developmental delay, extensive hemorrhage, and finally embryonic death.^29^

There are single nucleotide polymorphisms (SNPs) in *DICER1* and *DROSHA* genes which can affect their function or concentration. The location of rs3742330 SNP is in the 3ˊ‐untranslated region (UTR) of *DICER1* gene, which can affect the *DICER1* gene stability and expression.^30^ Our previous in silico study revealed that rs3742330C or G allele generated the novel binding sites for three miRNAs, that could trigger the mRNA degradation.^14^ In addition, the *DROSHA* rs6877842 polymorphism is located in the promoter region, which can affect the gene expression.

Several studies have found the relationship between *DICER1* and *DROSHA* polymorphisms and several diseases, but the published reports on their impacts on RSA are rare.^16^, ^19^, ^24^

Contrary to the results of current study, in their first investigation, Jung et al (2014) found that the combination of GG/TC + CC genotypes of *DICER1* rs3742330/*DROSHA* rs10719 polymorphisms was associated with increased idiopathic recurrent pregnancy loss in Korea. Indeed, they observed the effect of the combination of *RAN* rs14035 or *XPO5* rs11077 with *DICER1* rs3742330 on this disorder.^20^

Similarly, Fu et al found no relationship between unexplained recurrent spontaneous abortion (URSA) and *DICER1*, *DROSHA* and *RAN* polymorphisms alone. But in concordance with Jung et al’s study, they showed that *DICER1* rs3742330/DROSHA rs10719 GG/TC + TT combinations were associated with a higher URSA risk.^21^

Moreover, several studies have evaluated the possible effects of miRNA machinery gene polymorphisms on other pregnancy complications. Rah et al found no relation between *DICER1*, *DROSHA,* and *RAN* polymorphisms and primary ovarian insufficiency, but they observed a higher frequency of *XPO5* rs2257082 T allele in this disorder.^23^

Rezaei et al found the association between the *DROSHA* rs10719 (TC genotype) but not rs6877842 polymorphism and PE susceptibility in Iran.^24^ In another study, they reported the impact of placental *DROSHA* rs10719 polymorphism on PE risk in the recessive model. Indeed, the combination of CC/GG genotypes of *DROSHA* rs10719 and rs6877842 polymorphisms was associated with a higher risk of PE. The expression of *DROSHA* gene was downregulated in the placenta of PE women and those with rs10719CC genotype.^15^

In their study, Eskandari et al demonstrated the effect of placental but not maternal *DICER1* rs3742330 polymorphism on PE and PE severity. Indeed, they showed a decrease in mRNA expression of *DICER1* gene in the placenta of women with rs3742330 AG + GG genotypes.^14^ Recently, the association between *DICER1* rs13078 TA genotype and a higher risk of gestational hypertension has been reported by Huang et al^31^

Although the important role of miRNA machinery biogenesis enzymes in various biological processes has been established, the studies which evaluated the effects of each variant on expression or function of its corresponding enzyme are limited. Therefore, further studies are necessary to assess the effects of genetic variants in enzymes of this process on pregnancy disorders.

This study has some possible limitations which need to be pointed out. First, our data from placental pathology or immunologic parameters were not complete to analyze in association with the variants. Second, the relative small sample size could affect our results, especially for those with marginal *P*‐values. Third, if we could analyze these variants in aborted fetus as well as the mRNA and protein expression levels of Dicer1 and Drosha, our findings become more valuable.

In conclusion, the results of present study showed the protective role of *DICER1* rs3742330 polymorphism in RSA risk. However, there was no relationship between *DROSHA* rs6877842 polymorphism and RSA. The combination of *DICER1* rs3742330 AG and *DROSHA* rs6877842 GC genotypes and G‐G haplotype was associated with a reduced RSA risk; however, AA‐GG combined genotypes and A‐G haplotype could lead to an increased RSA risk.

## CONFLICT OF INTEREST

The authors declare no conflict of interest.
